# Evaluation of survivin immunoexpression in the differentiation of high- and low-grade breast ductal carcinoma *in situ*

**DOI:** 10.1590/S1679-45082018AO4065

**Published:** 2018-04-06

**Authors:** Milca Cezar Chade, Sebastião Piato, Maria Antonieta Longo Galvão, José Mendes Aldrighi, Rômulo Negrini, Evandro Falaci Mateus, Enio Martins Medeiros

**Affiliations:** 1 Faculdade de Ciências Médicas, Santa Casa de São Paulo, São Paulo, SP, Brazil; 2 Hospital Israelita Albert Einstein, São Paulo, SP, Brazil

**Keywords:** Inhibitor of apoptosis proteins, Carcinoma, intraductal, noninfiltrating, Biomarkers, tumor, Neoplasm proteins, Breast neoplasms, Proteínas inibidoras de apoptose, Carcinoma intraductal não infiltrante, Biomarcadores tumorais, Proteínas de neoplasias, Neoplasias da mama

## Abstract

**Objective:**

To evaluate the expression of survivin protein in low- and high-grade ductal carcinoma *in situ*.

**Methods:**

Breast tissue fragments obtained by incisional biopsy and surgical procedures of 37 women with ductal carcinoma *in situ* of the breast were subdivided into two groups: Group A, composed of women with low-grade ductal carcinoma *in situ*, and Group B, women with high-grade ductal carcinoma *in situ*. Survivin protein expression test was performed by immunohistochemistry, using a monoclonal antibody clone I2C4. The criterion to evaluate survivin immunoexpression was based on the percentage of neoplastic cells that presented brown-gold staining. This criterion was positive when the percentage of stained cells was ≥10%.

**Results:**

The survivin protein was expressed in 22 out of 24 cases of high-grade ductal carcinoma *in situ* (78%), whereas, in Group A, of low-grade ductal carcinoma *in situ* (n=13), it was positive in only 6 cases (21.40%; p=0.004).

**Conclusion:**

The frequency of expression of survivin was significantly higher in the group of patients with high-grade ductal carcinoma *in situ* compared to those in the low-grade ductal carcinoma *in situ* group.

## RESUMO

**Objetivo:**

Avaliar a imunoexpressão da proteína survivina nos carcinomas ductais *in situ* de mama de baixo e de alto graus.

**Métodos:**

Fragmentos de tecido mamários obtidos por biópsia incisional e procedimentos cirúrgicos de 37 mulheres acometidas por carcinoma ductal *in situ* de mama foram subdivididos em dois grupos: Grupo A, formado por mulheres com carcinoma ductal *in situ* de baixo grau; e Grupo B, por mulheres com carcinoma ductal *in situ* de alto grau. A pesquisa de expressão da proteína survivina foi realizada pela técnica de imuno-histoquímica, utilizando-se anticorpo monoclonal clone I2C4. O critério de avaliação da imunoexpressão da survivina baseou-se na percentagem de células neoplásicas que apresentava coloração castanho-dourada. Considerouse tal critério positivo quando a percentagem de células apresentasse marcação ≥10%.

**Resultados:**

A proteína survivina apresentou-se expressa em 22 dos 24 casos de carcinoma ductal *in situ* de alto grau (78%), enquanto no Grupo A, de carcinoma ductal *in situ* de baixo grau (n=13), apresentou-se positiva em apenas 6 casos (21,40%; p=0,004).

**Conclusão:**

O índice de frequência de expressão da survivina foi significativamente mais elevado no grupo de pacientes com carcinoma ductal *in situ* de alto grau, quando comparado às do grupo com carcinoma ductal *in situ* de baixo grau.

## INTRODUCTION

Breast cancer is the second most frequent type of neoplasm among women,^(^ ^1^ ^)^ representing approximately 22% of new cases diagnosed every year. In 2016, breast cancer incidence in the United States was of 231,840 cases, and 60,290 (21.7%) were diagnosed as ductal carcinoma *in situ* (DCIS).^(^ ^2^ ^)^ In some series of non-palpable tumors, detected by mammography in screening programs, up to 45% of cases were DCIS.^(^ ^3^ ^–^ ^5^ ^)^

It is noteworthy that, when left untreated, DCIS poses a risk between 30 and 50% of progressing to invasive carcinoma within 10 years.^(^ ^6^ ^,^ ^7^ ^)^ However, it is not yet clear which forms of DCIS lesions progress to an invasive disease or have an indolent development.

A better molecular and histopathological DCIS characterization can bring additional information to evaluate the prognosis of the disease, and allows customizing an appropriate treatment for each patient. There have been advancements in molecular studies for the assessment of risk and progression of premalignant diseases; however, they are still quite modest in clinical practice.^(^ ^6^ ^–^ ^8^ ^)^

There is evidence that the activity mentioned is significantly more marked in DCIS lesions than in invasive carcinoma.^(^ ^9^ ^)^ When comparing DCIS, lowgrade lesions present higher cell apoptosis rate than high-grade.^(^ ^9^ ^)^

Considering survivin protein has important antiapoptotic properties, some researchers have conducted studies to correlate its expression to aggressiveness of breast cancer.^(^ ^10^ ^–^ ^15^ ^)^

The antiapoptotic action of survivin can occur through the direct inhibition of effector caspases 3 and 7 and of initiator caspase 9, which play a relevant role in the mechanism of programmed cell death.^(^ ^16^ ^)^ Another survivin antiapoptotic action consists of antagonizing the activity of the second mitochondria-derived activator of caspase/direct inhibitor of apoptosisbinding protein with low pI (Smac/DIABLO). This protein, released from the mitochondria, binds to and removes inhibitors of apoptosis protein (IAP) from its inhibitory binds to caspases, thus promoting apoptosis. Thus, survivin, by inhibiting caspase activation, would increase cell survival, both directly and/or via Smac/DIABLO.^(^ ^16^ ^)^

Regarding the expression of survivin protein in neoplastic cells, Youssef et al.,^(^ ^17^ ^)^ observed an inverse correlation with the size of the primary tumor; in addition, the expression of estrogen and progesterone receptors is directly proportional to size of neoplasm and survivin expression. Similarly, other clinical trials demonstrated that survivin is associated to a poor prognosis and lower rates of disease-free survival.^(^ ^15^ ^,^ ^18^ ^)^

Some retrospective studies on breast cancer showed that survivin protein is an important marker of cancer aggressiveness and poor prognosis, leading to decreased overall survival.^(^ ^19^ ^,^ ^20^ ^)^ The studies on messenger RNA (mRNA) microarray are consistent with these results, also identifying survivin as a risk factor associated to breast cancer.^(^ ^21^ ^,^ ^22^ ^)^

In a study about the correlation between survivin expression and prognosis, conducted with 167 women with breast cancer stages I, II, and II, Tanaka et al.,^(^ ^23^ ^)^ found survivin expression in 70% (118) of tumors. They saw that survivin expression was the most consistent prognostic factor in comparison to other clinicopathological prognostic characteristics, including tumor size, clinical stage, lymphatic involvement, hormone receptors and histological type.

In an investigation published in 2007, Yamashita et al.,^(^ ^10^ ^)^ reported survivin is an indicator of recurrence risk for early stage breast cancer. In 2008, Okumura et al.,^(^ ^24^ ^)^ published the results of a study about survivin expression, which included 52 cases of pure DCIS and 28 cases of DCIS with foci of microinvasion (DCIS-MI), and showed expression of this protein was significantly higher in the DCIS-MI cases than in those with a sample of only DCIS.

A comparative study conducted in Brazil found a significantly higher expression of survivin in specimens of triple-negative breast carcinoma, which is generally highly aggressive, than in specimens of luminal A breast carcinoma, which is notably known as less aggresive.^(^ ^25^ ^)^

## OBJECTIVE

To evaluate survivin protein expression in low-grade and high-grade breast ductal carcinoma *in situ*.

## METHODS

For this study, we selected 37 fragments of surgical specimens or biopsy material from patients with breast DCIS. All patients were treated in the Mastology Sector -Department of Gynecology and Obstetrics, between 2014 and 2016. Only samples with low-and high-grade DCIS were selected, with no associated invasive lesions and no previous treatment.

The study was previously approved by the hospital Research Ethics Committee for complying with ethical principles, Resolution 466/96 from the National Health Council/Ministry of Health, and with additional internal norms, under protocol number 1674151, CAAE: 57764416.4.0000.5479.

The specimens were classified as Groups A and B, according to the results of histopathological exams carried out at the Department of Pathology. Group A included 13 cases of low-grade DCIS, and Group B had 24 cases of high-grade DCIS.

Specimen selection for both groups included the following exclusion criteria: association of neoplasm and pregnancy and nursing; previous chemotherapy, hormone therapy or radiation therapy; and inappropriate material.

To confirm the diagnosis of both forms of DCIS, the same examiner reviewed the histological exams. Intermediate-grade DCIS cases were also excluded.

Initially, each fragment was embedded in a paraffin block. From each block, a 4µm thick histological section was obtained using a rotating microtome. The sections were stained with hematoxylin-eosin (HE), and assessed by light optical microscopy. Characterization of histological types as high- and low-grade DCIS was done according to guidelines of the Brazilian Society of Pathology. Survivin protein expression was analyzed by immunohistochemistry, in 4µm thick sections, using a lyophilized anti-human mouse monoclonal antibody, clone I2C4, IgG2a Kappa immunoglobulinisotype class. Each kit contained 1mL and was diluted to 1/50. The pH was 6.0, and recovery was done through microwaves and an incubation period of 15 minutes.

**Figure 1:**
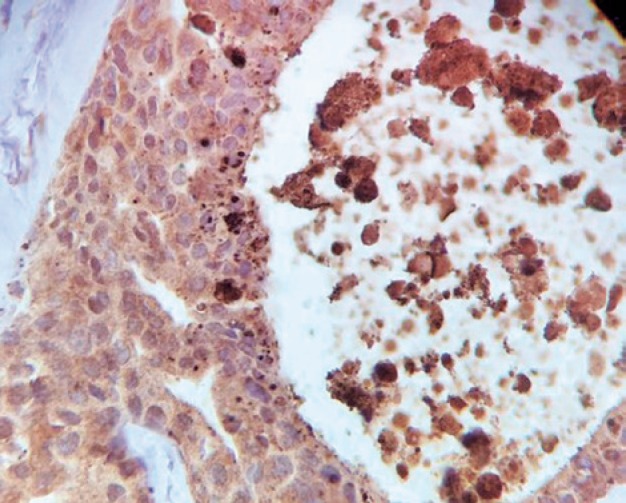
Breast ductal carcinoma *in situ* cell nuclei and cytoplasm stained brownish gold by antisurvivin antibody (400x)

Survivin immunoexpression through breast DCIS epithelial cells occurs in the nuclei and in the cytoplasm, as shown in figure 1.

**Figure 2:**
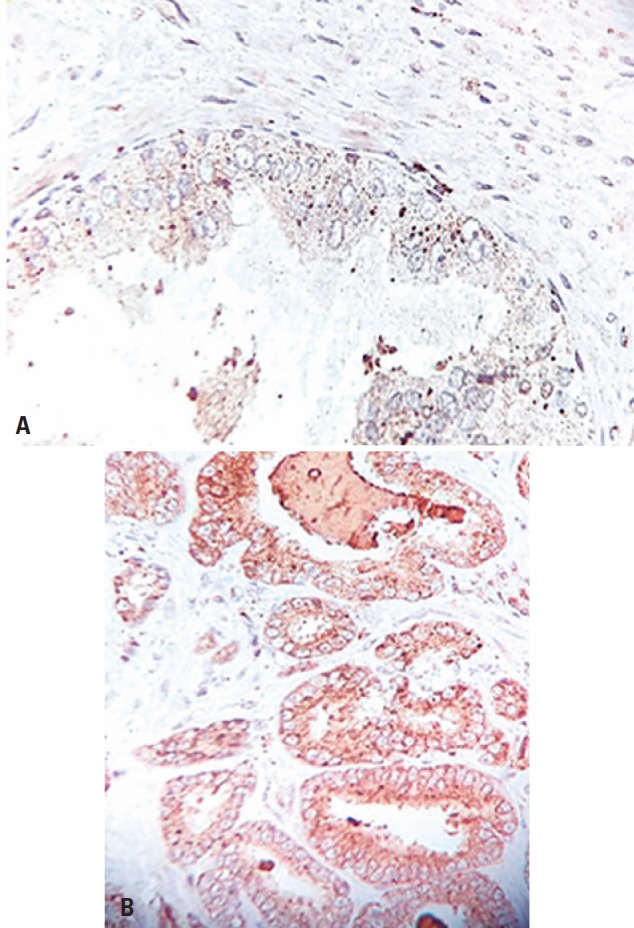
(A) Prostate tumor sample considered as negative control for anti-survivin reaction (400x). (B) Prostate tumor sample considered as positive control for antisurvivin reaction (400x)

As per the manufacturer's recommendations, to evaluate survivin immunoexpression, we used, as a positive control, samples of prostate tissue fragments, in which the brownish gold stains are intense in the nucleus and cytoplasm. In figure 2A, we can see a histological section of prostate tumor with negative control; and in figure 2B, we find a positive control.

Immunohistochemical reactions were assessed by two independent evaluators using a Nikon Eclipse E400 microscope with two binocular heads.

The evaluation criteria for survivin expression was based on the percentage of neoplastic cells that presented a brownish gold staining in the nucleus or cytoplasm. The criterion was considered positive when ≥10% of sample cells were stained.^(^ ^25^ ^)^

To further stratify the positivity and negativity grades of samples marked by the antisurvivin antibody, these samples was subdivided as follows (Table1).

**Table 1:** Positivity and negativity grade stratification of samples marked by survivin

Survivin	
Negative – 0	Positive – 2
0 and 1 if ≤10%	2 and 3 if >10%

In all slides considered positive, nucleus and cytoplasm were equally positive.

**Figure 3:**
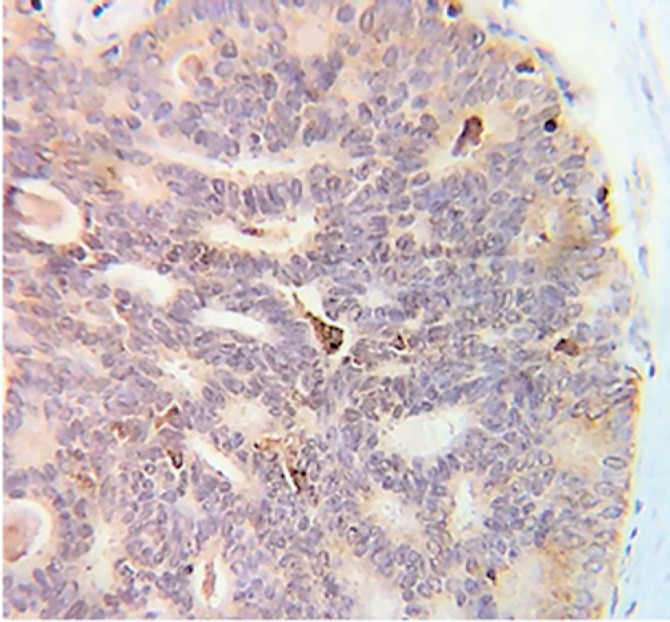
Sample of ductal carcinoma *in situ* considered negative for anti-survivin reaction (400x)

**Figure 4:**
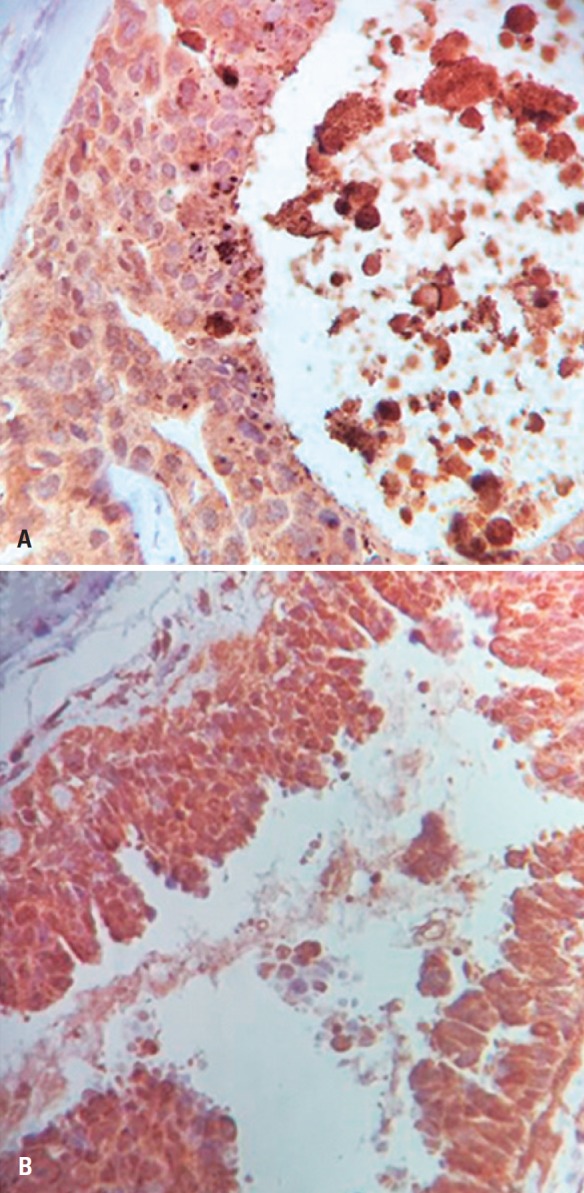
(A) Sample of high-grade ductal carcinoma *in situ* (with comedonecrosis) considered positive for anti-survivin reaction. (B) Sample of high-grade ductal carcinoma *in situ* considered positive for anti-survivin reaction (400 x).

Figure 3 shows a histological section of DCIS tissue samples considered negative for antisurvivin reaction, and figure 4 shows a premalignant tissue histological section considered positive.

### Statistical analysis

For the association between the variable survivin and Groups A and B, we applied Fisher's exact test. Significance level was set at 5% (0.050) for statistical tests. A statistically significant difference was characterized when the calculated significance value (p) was <5% (0.05). A calculated significance value (p) ≥5% (0.050) indicated a non-statistically significant difference or relation. We also used a likelihood ratio test to assess data stratification.

An MS-Excel electronic spreadsheet, version MS-Office 2013, was used to organize the data, and the IBM Statistical Package for the Social Sciences (SPSS), version 23.0 was used to obtain the results.

## RESULTS

Survivin protein was positive in 22 out of 24 high-grade DCIS cases (78.6%), whereas in the group of 13 low-grade DCIS cases, survivin was positive in 6 of them (21.4%). In 77.8% of cases survivin was negative, and DCIS was low-grade (Table 2).

**Table 2:** Comparison of survivin positive and negative expression in high- and low-grade ductal carcinoma *in situ*

Survivin (positive-negative)	Nuclear grade classification n (%)	
	High	Low
Positive	6 (21.40)	22 (78.60)
Negative	7 (77.80)	2 (22.20)

Fisher's exact test: p=0.004.

The statistical analysis showed a significantly elevated survivin expression in high-grade DCIS as compared to low-grade lesions (p=0.004).

When comparing survivin positive and negative expression, subdivided into zero, 1, 2, and 3 in Groups A and B, we applied the likelihood ratio test to verify a possible difference between the four categories of the variable survivin for the variable nuclear grade classification.

The results in table 3 show that survivin expression was significantly more elevated in high-grade DCIS as compared to low-grade DCIS, when classifying the positivity grade (p=0.001). Among the lesions with negative expression, those classified as zero were low-grade in 100% of cases; whereas those classified as 1 were observed in 60%. And the high-grade lesions, classified as 3, were strongly positive in 84% of cases.

**Table 3:** Comparison between survivin positive and negative expression, subdivided into zero, 1, 2 and 3, in high- and low-grade ductal carcinoma *in situ*

Survivin	Nuclear grade classification n (%)	
	Low	High
0 negative	4 (100)	0
1 negative	3 (60.00)	2 (40.00)
2 positive	2 (66.70)	1 (33.30)
3 positive	4 (16.00)	21 (84.00)

Fisher's exact test: p=0.001.

## DISCUSSION

Proposals about the procedures used in breast DCIS treatment greatly vary, ranging from insufficient to excessive. To reach a consensus regarding the appropriate treatment for low- and high-grade DCIS, several studies related to predictive factors have been developed.

The classic prognostic factors of breast DCIS do not accurately predict local recurrence. The discovery of molecular biomarkers has played an important role in prognosis and decisions about treatment, including conservative therapies, mastectomy, radiation and hormone therapy. However, it is thought that the use of biomarkers is not enough to establish an ideal DCIS management. Therefore, a new predictive strategy has been recently proposed, which consists of a DCIS recurrence and prognosis score, as a modified Oncotype Dx (Genomic Health, Redwood City, CA, USA). This proposal undoubtedly offers advancements that will allow an even better patient selection, especially for adjuvant therapies, making way for a individualized treatment plan.^(^ ^26^ ^)^

Davis et al.,^(^ ^27^ ^)^ analyzed a biomarker panel (estrogen receptor, HER-2, Ki67, p53, cyclin D1, COX-2, caveolin-1, survivin, and PPAR-g) and a DCIS clinical and histological factor panel to determine associations with recurrence of disease. The variables analyzed in the study with 70 patients were age, tumor size, margin, grades of *in situ* tumors, presence of necrosis, and histological type.

The proponents of the abovementioned score included in their algorithm the survivin gene, among other neoplasm-related genes, considering many studies that highlighted the role of survivin in carcinomas from different organs, including breast,^(^ ^8^ ^,^ ^23^ ^)^ regarding carcinogenesis, prognosis and survival.

There are few investigations about survivin protein, despite the evidence of its relevant role in breast carcinogenesis and several studies recognizing it is an effective prognostic and disease-free survival marker. Scarce research is particularly noticeable regarding the lesion that precedes invasive breast cancer, *i.e* We believe this is very relevant considering survivin may allow a target therapy based on its inhibitory action.

In a study published in 2012 that included lymphoblastic leukemia patients, Tyner et al.,^(^ ^28^ ^)^ observed that sepantronium bromide (YM-155) was able to inhibit survivin action, thus reducing lymphoblast activity. Kumar et al.,^(^ ^29^ ^)^ found YM-155 reverted cisplatin resistance in patients with head and neck carcinoma, making chemotherapy more effective. In a prospective study with multiple myeloma patients, de Haart et al.,^(^ ^30^ ^)^ observed a prolonged remission of the disease with the use of YM-155.

## CONCLUSION

Survivin protein immunoexpression was significantly more elevated in epithelial cells of high-grade breast ductal carcinoma *in situ* as compared to low-grade lesions. There was a significant correlation between survivin immunoexpression and the differentiation between lowand high-grade ductal carcinoma *in situ*. We believe the results from this study, despite its relatively small sample, contribute to the current effort to develop new tools to improve the objective grade differentiation in ductal carcinoma *in situ*, leading to better management of ductal carcinoma *in situ* of the breast.
